# Cimifugin ameliorates imiquimod-induced psoriasis by inhibiting oxidative stress and inflammation via NF-κB/MAPK pathway

**DOI:** 10.1042/BSR20200471

**Published:** 2020-06-17

**Authors:** Aimin Liu, Wei Zhao, Buxin Zhang, Yuanhui Tu, Qingxing Wang, Jing Li

**Affiliations:** Department of Dermatology, Henan Province Hospital of Traditional Chinese Medicine, The Second Affiliated Hospital of Henan University of Chinese Medicine, Zhengzhou 450002, People's Republic of China

**Keywords:** pharmacology, psoriasis, oxidative stress, inflammation

## Abstract

Cimifugin is an important component of chromones in the dry roots of *Saposhikovia divaricata* for treating inflammatory diseases. However, the possible effect of cimifugin in psoriasis needs further investigation. This current work was designed to evaluate the effects of cimifugin in psoriasis *in vivo* and *in vitro*, and unravel the underlying molecular mechanism. Here, we used imiquimod (IMQ) or tumor necrosis factor (TNF)-α to induce a psoriasis-like model in mice or keratinocytes. Obviously, the results showed that cimifugin reduced epidermal hyperplasia, psoriasis area severity index (PASI) scores, ear thickness and histological psoriasiform lesions in IMQ-induced mice. The decreased levels of reduced glutathione (GSH), superoxide dismutase (SOD) and catalase (CAT), and the accumulation of malondialdehyde (MDA) in skin tissues by IMQ were attenuated by cimifugin. Furthermore, it was observed that cimifugin effectively reversed IMQ-induced up-regulation of proinflammatory cytokines, including TNF-α, IL-6, IL-1β, IL-17A, and IL-22. Mechanically, we noticed that cimifugin inhibited IMQ-activated phosphorylation of NF-κB (IκB and p65) and MAPK (JNK, ERK, and p38) signaling pathways. Similar alterations for oxidative stress and inflammation parameters were also detected in TNF-α-treated HaCaT cells. In addition, cimifugin-induced down-regulation of ICAM-1 were observed in TNF-α-treated cells. Altogether, our findings suggest that cimifugin protects against oxidative stress and inflammation in psoriasis-like pathogenesis by inactivating NF-κB/MAPK signaling pathway, which may develop a novel and effective drug for the therapy of psoriasis.

## Introduction

Psoriasis is a common chronic inflammatory disease related to autoimmune, which severely impairs the life quality of patients [1,2]. It is reported that the main pathological features of psoriasis are characterized by the dysregulation of cytokines and chemokines, infiltration of inflammatory cells, and hyperproliferation of keratinocytes, thus resulting in aberrant epidermal hyperplasia [1]. During psoriasis development, the increased expression of inflammatory cytokines, such as tumor necrosis factor (TNF)-α, interleukin (IL)-6, IL-17A, and IL-22 triggers uncontrolled inflammatory states and activates keratinocyte hyperproliferation [3,4]. In addition, increasing evidence suggest that oxidative stress may aggravate the psoriasis pathogenesis. The impaired antioxidant defense system affects lipid peroxidation, DNA modification, and inflammatory molecule secretion [5,6]. Recent studies suggest that topical agents or systemic treatments are commonly used to therapy psoriasis with different severities in clinical. The long-term treatment for psoriasis may cause a significant economic burden for patients [7]. The potential side effects are also demonstrated to be exited in the prolong usage. For example, cyclosporine has close implications in the increased occurrences for hypertension, renal dysfunction, and non-melanoma skin cancer [8]. Thus, it is necessary to elucidate the precise molecular mechanisms and open a novel insight for developing effective therapies of psoriasis.

The dry root of *Saposhikovia divaricata*, also called ‘Fang-Feng’, is a traditional Chinese herb that has anti-inflammatory and anti-allergic properties and is widely used as a drug in rheumatism, urticaria, and skin pruritus [9,10]. It is found that chromones are the major active ingredients in ‘Fang-Feng’, consisting of Prim-oglucosylcimifugin and cimifugin [11–13]. Previous studies have demonstrated that Prim-oglucosylcimifugin exerts anti-inflammation and analgesia effects, which may be converted into cimifugin *in vivo* [14,15]. Cimifugin is shown to inhibit allergic inflammatory responses by the modulation of tight junctions [16]. Recently, Han et al. suggest that cimifugin protects against the inflammatory cytokines production in an *in vitro* rheumatoid arthritis model [17]. Our previous work noticed that ‘Mafang Xijiao Dihuang Decoction’, including Fang-Feng component, ameliorated the psoriasis area severity index (PASI) index and suppressed the expression levels of IL-17 and VEGF in peripheral blood for psoriasis patients [18], indicating its potential pharmacological activities in inflammatory microenvironment. However, the precise mechanism in psoriasis remains to be further elucidated. Therefore, we speculated that cimifugin might attenuate the pathogenesis of psoriasis through inhibiting oxidative stress and inflammatory responses.

In the present study, the imiquimod (IMQ)-induced psoriasis-like mouse model and TNF-α-induced keratinocytes were employed to determine the effects of cimifugin *in vivo* and *in vitro*. In addition, the important factors associated with oxidative stress and inflammation were examined to further investigate the possible regulatory mechanism in psoriasis.

## Materials and methods

### Animal model

All animal care and experiments were conducted the Central Laboratory of Henan Province Hospital of Traditional Chinese Medicine, The second Affiliated Hospital of Henan University of Chinese Medicine. Ethical statement was approved by The Second Affiliated Hospital of Henan University of Chinese Medicine. The animal experimental procedures were performed in accord with the Guide for the Care and Use of Laboratory Animal. Male BALB/c mice (8–11 weeks) were housed in normal environment with free access to food and water. IMQ was commonly used to develop a psoriasis-like mouse model as previously reported [19]. For IMQ treatment, mice were shaved on the back skin and left ear, which were applied topically with IMQ (19030239; Mingxin Pharmaceutical Co., Ltd, Sichuan, China) at a dose of 62.5 mg daily per 5 cm^2^ for six consecutive days. The cimifugin (CIM; IC0410) was purchased from Solarbio (Beijing, China). The low dose (CIM-L) and high dose (CIM-H) groups of CIM were administrated with CIM (12.5 or 50 mg/kg/day) intragastrically 2 days before IMQ treatment until the sixth day of IMQ model. The mice in IMQ+vehicle group received the equal volume of saline intragastrically in line with the CIM administration. Control mice were just shaved without any drug treatment. After recording the PASI scores and ear thickness on seventh day, these animals were killed by intraperioneal injection with 200 mg/kg sodium pentobarbital.

### Hematoxylin and eosin (HE) staining

Skin tissues were embedded in paraffin and cut into 5-μm sections. Then hematoxylin (H8070; Solarbio) and eosin (A600190; Sangon, Shanghai, China) solutions were used to stain sections according to the standard method. Images were captured under a microscope (BX53; OLUMPUS, Tokyo, Japan) at ×200 magnification.

### Cell culture and CCK8 assay

Human immortalized keratinocytes (HaCaT) cell line was obtained from Procell (Wuhan, China) and cultured in a MEM medium with 15% fetal bovine serum (FBS) in a humidified atmosphere (5% CO_2_, 37°C). To mimic the psoriasis-like inflammation, HaCaT cells were treated with TNF-α (10 ng/ml) for 12 h.

Furthermore, CCK8 assay was performed to measure the effect of cimifugin with different concentrations on HaCaT cell proliferation. Cells were treated with cimifugin at 0, 0.01, 0.1, 1, or 10 μM for 24 h. Subsequently, cells were treated using CCK8 kits (KGA317; KeyGen, Nanjing, China), and the absorbance was determined at the wavelength of 450 nm. Finally, the optical concentrations of cimifugin (0.01, 0.1, and 1 μM) were chosen to add into the medium for 12 h prior to TNF-α stimulation. Then cell supernatants were collected for further examinations.

### Immunofluorescence

Immunofluorescence staining was conducted to evaluate the activation of NF-кB signaling pathway. After fixed in 4% paraformaldehyde, cells were blocked with goat serum and incubated with specific primary antibody against p65 (A2547, Abclonal, Wuhan, China) overnight at 4°C. Then Cy3-labeled goat anti-rabbit secondary antibody (A0516, Beyotime, Shanghai, China) was used to conjugate p65 primary antibody for 60 min at room temperature. After counterstaining with DAPI, the cell coverslips were imaged under the microscope at ×400 magnification.

### Measurement for oxidative stress factors

The commercial kits for malondialdehyde (MDA; A003-1), reduced glutathione (GSH; A006-2), total superoxide dismutase (SOD; A001-1), and catalase (CAT; A007-1) were purchased from Nanjing Jiancheng Bio Ins (Nanjing, China). The protein samples from skin tissues or cell supernatants were extracted and used to determine the contents of aforementioned oxidative stress factors using available kits following manufacturer's instructions.

### Enzyme linked immunosorbent assay

The protein extracts from skin tissues or cell supernatants were prepared to determine the concentrations of pro-inflammatory cytokines using enzyme linked immunosorbent assay (ELISA) kits as described by manufacturer's protocols. The ELISA kits for mouse TNF-α (EK282/3), IL-1β (EK201B/3), IL-6 (EK206/3), IL-17A (EK217/2), IL-22 (EK222/2), human ICAM-1 (EK189), IL-6 (EK106/2), and IL-1β (EK101BHS) were purchased from Multi Sciences (Hangzhou, China).

### Quantitative real-time PCR

The total RNAs from mouse skin tissues were isolated and reverse-transcribed into cDNA templates. To amplify the target genes, the specific primers (5′–3′) were designed as follows: TNF-α (forward CAGGCGGTGCCTATGTCTCA and reverse GCTCCTCCACTTGGTGGTTT), IL-6 (forward ATGGCAATTCTGATTGTATG and reverse GACTCTGGCTTTGTCTTTCT), IL-1β (forward CTCAACTGTGAAATGCCACC and reverse GAGTGATACTGCCTGCCTGA), IL-17A (forward AAACACTGAGGCCAAGGAC and reverse CGTGGAACGGTTGAGGTAG), IL-22 (forward GACAGGTTCCAGCCCTACAT and reverse CAGCCTTCTGACATTCTTCT), and GAPDH (forward TGTTCCTACCCCCAATGTGTCCGTC and reverse CTGGTCCTCAGTGTAGCCCAAGATG). The analysis of quantitative real-time PCR (qPCR) was performed using SYBR Green reagent (SY1020, Solarbio). Finally, 2^−ΔΔCT^ method was used to calculate relative gene expression.

### Western blot

Total proteins from mouse skin tissues or HaCaT cells were prepared to separate using SDS/PAGE and then transferred onto PVDF membranes (IPVH00010; Millipore, Billerica, MA, U.S.A.). The membranes were incubated with primary antibodies overnight at 4°C, including p-JNK antibody (#4668; CST, Danvers, MA, U.S.A.), JNK antibody (#9252; CST), p-ERK antibody (#4370; CST), ERK (#4695; CST), p-IкB (#2859; CST), IкB (#9242; CST), p-p65 (#3033; CST), p65 antibody (#8242; CST), p-p38 antibody (bs-0636R; Bioss, Beijing, China), p38 antibody (bs-0637R; Bioss), and GAPDH antibody (60004-1-Ig; Proteintech, Wuhan, China). Then corresponding secondary HRP-conjugated goat anti-rabbit antibody (SE134; Solarbio) or goat anti-mouse antibody (SE131; Solarbio) was prepared to the membranes for 1 h at 37°C. After visualized using ECL reagent (PE0010; Solarbio), the immunoblots were imaged and the protein intensity was measured by Gel-Pro-Analyzer Software.

### Statistical analysis

All statistics were shown as mean ± SD, and analyzed using GraphPad Prism Software. One-way ANOVA followed Bonferroni's test was conducted to determine statistical differences among multiple groups. *P*<0.05 was considered as significant in statistics.

## Results

### Cimifugin ameliorated psoriasis symptoms in IMQ-induced psoriasis-like mice

**Figure 1:**
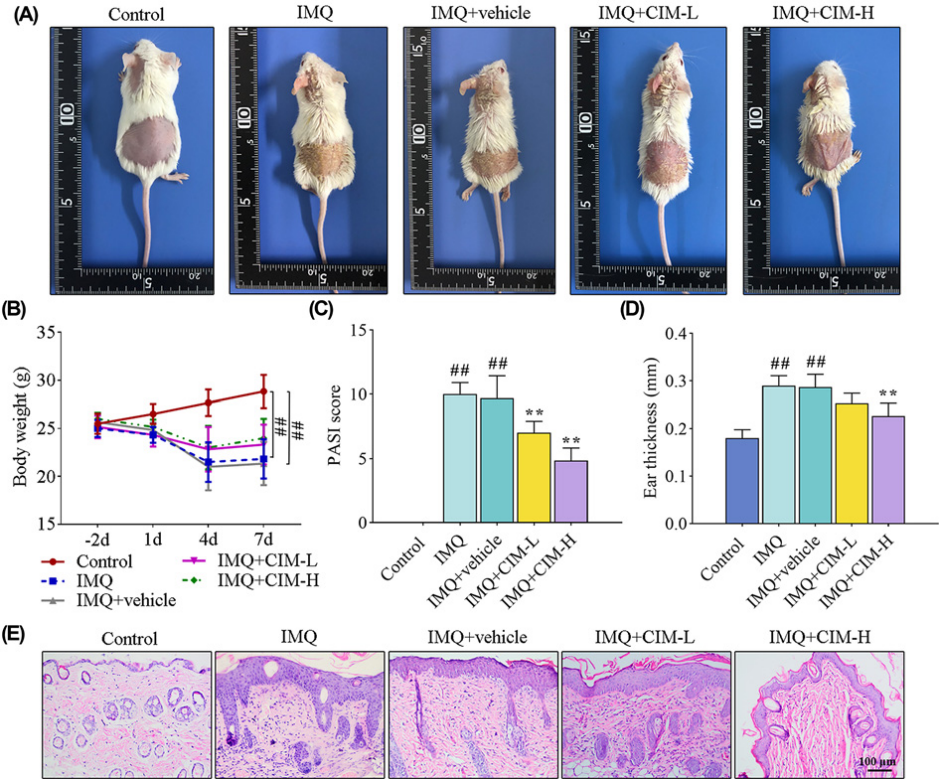
Cimifugin ameliorated psoriasis symptoms in IMQ-induced psoriasis-like mice (**A**) Images for the psoriatic appearance on mouse skin. (**B**–**D**) The body weight (**B**), PASI score (**C**), and ear thickness (**D**) were measured to assess the skin lesions. (**E**) Histological changes of mouse back skin tissues were determined by HE staining. CIM-L, low dose of cimifugin; CIM-H, high dose of cimifugin. ## *P*<0.01, compared with Control group; ** *P*<0.01, compared with IMQ+vehicle group.

To evaluate the effect of cimifugin on IMQ-induced psoriasis, IMQ was topically applied on the shaven back and left ear skin for consecutive 6 days. As shown in Figure 1A, IMQ treatment caused severe psoriatic phenotypes (scaling and erythema) on the skin (Figure 1A), which could be attenuated by cimifugin administration. The IMQ-treated mice became thinner than the normal mice (Figure 1B). However, in comparison with IMQ-induced psoriasis-like mice, no significant alteration of body weight in mice treated with CIM (Figure 1B). In addition, the PASI scores and ear thickness were apparently increased in IMQ-induced mice (Figure 1C,D). However, cimifugin treatment elicited a reduction in PASI scores and ear thickness (Figure 1C,D). Consistent with the macroscopic appearance changes, histological results of the skin indicated that IMQ significantly thickened epidermis, and cimifugin administration inhibited the epidermal hyperplasia (Figure 1E). Together, cimifugin had protective effects on IMQ-induced psoriasis-like mice.

### Cimifugin inhibited oxidative stress and inflammation in IMQ-induced mice

**Figure 2:**
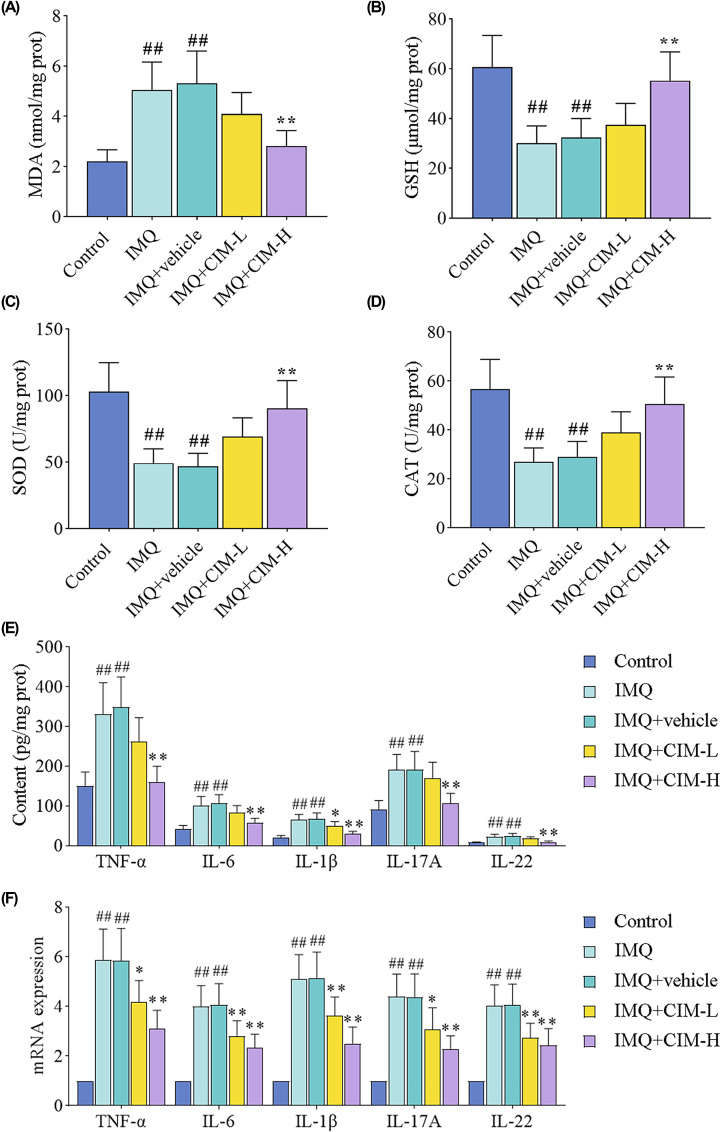
Cimifugin inhibited oxidative stress and inflammation in IMQ-induced mice (**A**–**D**) The contents of MDA (**A**), GSH (**B**), SOD (**C**), CAT (**D**) in back skin tissues were measured. (**E,F**) The protein (**E**) and mRNA (**F**) changes of pro-inflammatory cytokines (TNF-α, IL-6, IL-1β, IL-17A, and IL-22) were detected using ELISA and qRT-PCR. CIM-L, low dose of cimifugin; CIM-H, high dose of cimifugin. ## *P*<0.01, compared with Control group; * *P*<0.05, ** *P*<0.01, compared with IMQ+vehicle group.

We measured the levels of MDA, GSH, SOD, and CAT in back skin tissues. As shown in Figure 2A, higher levels of MDA in IMQ-treated mice were observed as compared with the control mice, which were decreased by cimifugin at the dose of 50 mg/kg. Topical administration with IMQ caused a significant reduction in the anti-oxidative biomarker levels (GSH, SOD, and CAT), and cimifugin treatment with high dose reversed the decreased levels near to normal levels (Figure 2B–D). Psoriasis is a chronic inflammatory skin disease, thus the changes of inflammatory cytokines (TNF-α, IL-6, IL-1β, IL-17A, and IL-22) were further examined in Figure 2E,F. The higher concentrations of inflammatory cytokines in IMQ-induced mice were significantly reduced by high dose of cimifugin (Figure 2E). Similarly, at the mRNA level, we noticed that cimifugin administration at 12.5 and 50 mg/kg both inhibited the increased levels of corresponding mRNAs for inflammatory factors (Figure 2F). These data indicated that cimifugin could inhibit IMQ-induced oxidative stress and inflammation.

### Cimifugin suppressed the activation of NF-кB and MAPK signaling pathways in IMQ-induced mice

**Figure 3:**
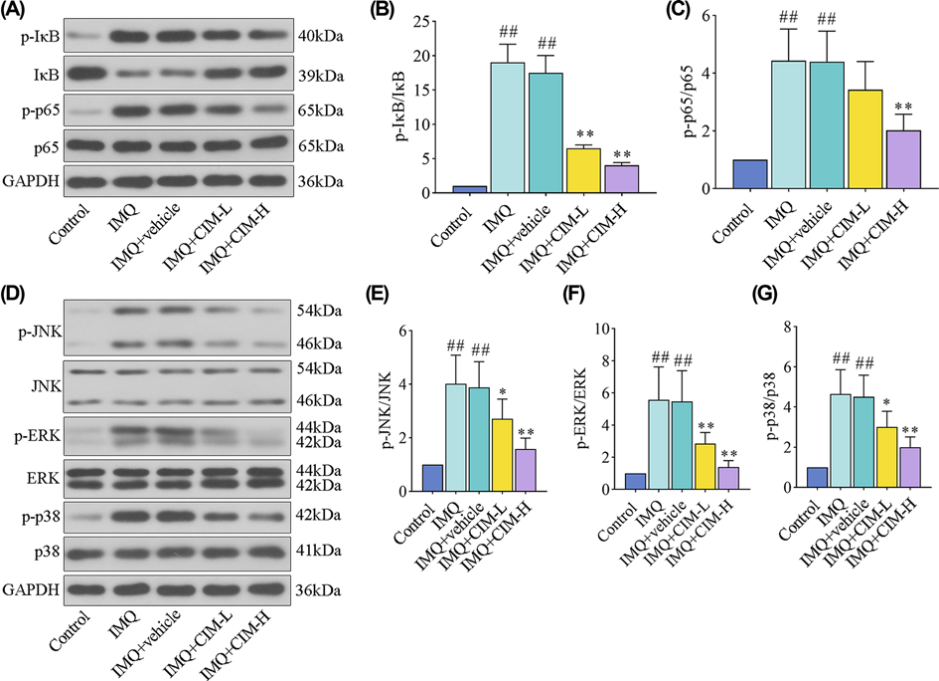
Cimifugin suppressed the activation of NF-кB and MAPK signaling pathways in IMQ-induced mice (**A**–**C**) The expression levels of p-IкB, IкB, p-p65, and p65 proteins for NF-кB signaling pathway were detected by western blot. (**D**–**G**) The expression levels of p-JNK, JNK, p-ERK, ERK, p-p38, and p38 proteins for MAPK signaling pathway were tested by western blot. CIM-L, low dose of cimifugin; CIM-H, high dose of cimifugin. ## *P*<0.01, compared with Control group; * *P*<0.05, ** *P*<0.01, compared with IMQ+vehicle group.

It is reported that NF-κB and MAPK signaling cascades are associated with oxidative stress and inflammatory response. Thus, to elucidate the involvement of probable signaling pathways in cimifugin treatment, the key factors were detected in IMQ-induced mice. The results from Figure 3 showed that IMQ promoted the phosphorylation of IκB, p65 NF-κB (Figure 3A–C), and three MAPKs (Figure 3D–G) including JNK, ERK, and p38 MAPK. However, upon the treatment of cimifugin, the alterations of NF-κB and MAPK signaling pathways in IMQ-induced mice were significantly reversed (Figure 3). Therefore, these results suggested that cimifugin treatment might inactivate the NF-κB and MAPK signaling pathways to impede oxidative stress and inflammatory response.

### Cimifugin attenuated TNF-α-induced oxidative stress and inflammation in HaCaT cells

**Figure 4:**
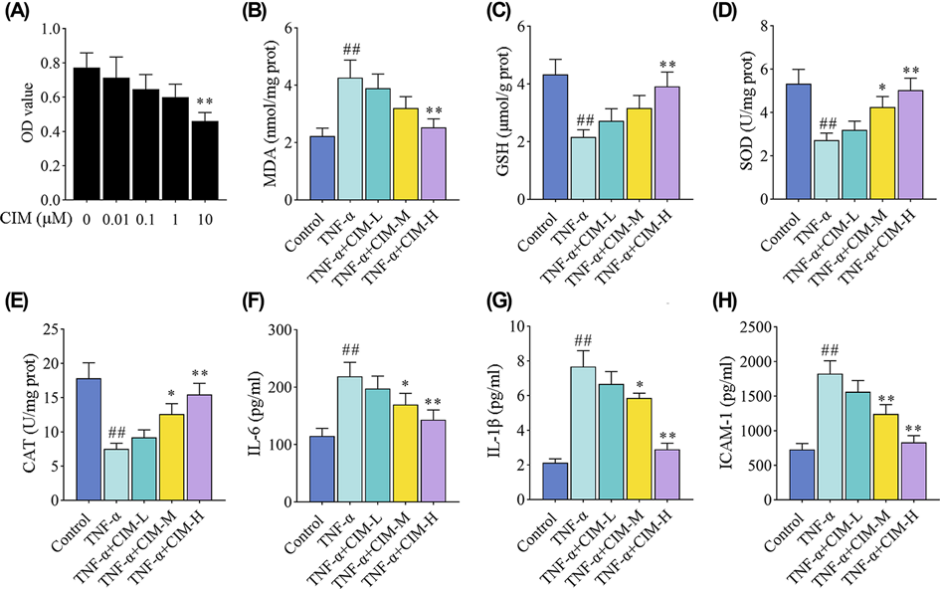
Cimifugin attenuated TNF-α-induced oxidative stress and inflammation in HaCaT cells (**A**) CCK8 assay was performed to measure the cell viability after the treatment of cimifugin with different concentrations. (**B**–**E**) The contents of MDA (**B**), GSH (**C**), SOD (**D**), CAT (**E**) in HaCaT cells were detected. (**F–H**) The protein changes of pro-inflammatory cytokines (IL-6, IL-1β, and ICAM-1) were determined by ELISA. CIM-L, low dose of cimifugin; CIM-M, medium dose of cimifugin; CIM-H, high dose of cimifugin. ## *P*<0.01, compared with Control cells; * *P*<0.05, ** *P*<0.01, compared with TNF-α-treated cells.

To further investigate the effects of cimifugin on psoriasis-like symptoms, the anti-oxidative and anti-inflammatory properties were also tested *in vitro*. CCK8 assay was first performed to assess HaCaT cell viability with different concentrations of cimifugin. The results in Figure 4A indicated that the concentrations of cimifugin up to 1 μM led no significant reduction in the viable HaCaT cells. Thus, the safe concentrations of cimifugin (0.01, 0.1, and 1 μM) were employed to treat HaCaT cells. As shown in Figure 4B–E, the increased levels of MDA (Figure 4B), as well as decreased levels of GSH, SOD, and CAT (Figure 4C–E) in HaCaT cells under TNF-α stimulation were reversed by cimifugin treatment. We also observed that the secretion of pro-inflammatory cytokines (IL-6, IL-1β, and ICAM-1) in epidermal keratinocytes was promoted by TNF-α, and blocked by cimifugin treatment (Figure 4F–H). Together, these results suggested that cimifugin exhibited anti-oxidative and anti-inflammatory properties of epidermal keratinocytes.

### Cimifugin suppressed TNF-α-induced activation of NF-кB and MAPK signaling pathways in HaCaT cells

**Figure 5:**
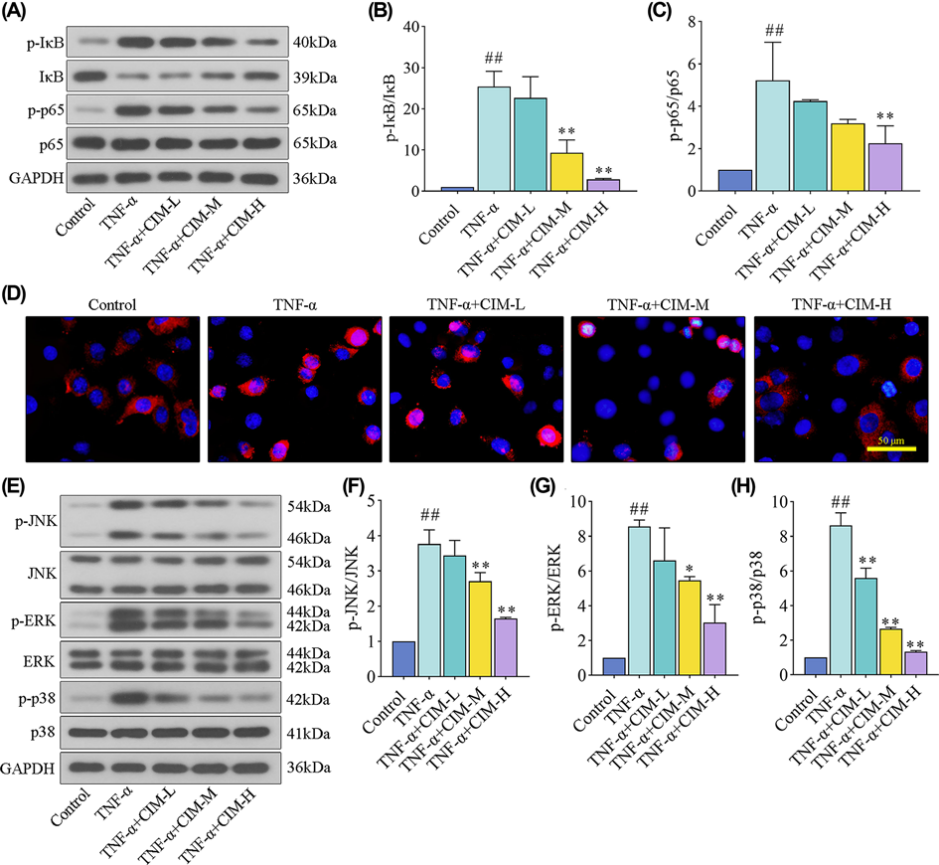
Cimifugin suppressed TNF-α-induced activation of NF-кB and MAPK signaling pathways in HaCaT cells (**A**–**C**) The expression levels of p-IкB, IкB, p-p65, and p65 proteins for NF-кB signaling pathway were detected by western blot. (**D**) The activation of p65 was evaluated using immnofluorescence staining. (**E**–**H**) The expression levels of p-JNK, JNK, p-ERK, ERK, p-p38, and p38 proteins for MAPK signaling pathway were tested by western blot. CIM-L, low dose of cimifugin; CIM-M, medium dose of cimifugin; CIM-H, high dose of cimifugin. ## *P*<0.01, compared with Control cells; * *P*<0.05, ** *P*<0.01, compared with TNF-α-treated cells.

Finally, the alterations of NF-κB and MAPK signaling pathways were assessed in TNF-α-treated HaCaT cells. As shown in Figure 5A–C, TNF-α stimulation phosphorylated the levels of IκB and p65 NF-κB proteins, whereas cimifugin rescued these protein alterations. Immunofluorescent images showed that the translocation of p65 NF-κB from cytoplasm into nucleus was blocked by cimifugin treatment in HaCaT cells exposed to TNF-α (Figure 5D). Furthermore, it was obviously observed that the phosphorylated levels of JNK, ERK, and p38 MAPK proteins were reduced by cimifugin administration concentration dependently in TNF-α-treated HaCaT cells (Figure 5E–H). Totally, the data showed that cimifugin inhibited oxidative stress and inflammation by inactivating NF-κB and MAPK signaling pathways.

## Discussion

Psoriasis is a chronic relapsing skin disease with nearly 2% prevalence rate worldwide [20]. The long-term therapy may cause a significant economic and mental burden. A number of co-morbidities are shown to be associated with psoriasis, such as cardiometabolic diseases, and depression [8]. Therefore, exploring an effective therapy for psoriasis has critical significances in the basic and clinical research. In the present study, our data showed that cimifugin suppressed the increases of PASI scores and ear thickness, attenuated the epidermal hyperplasia, and induced smooth epidermis in IMQ-induced mice. At molecular level, we found that IMQ- or TNF-α-mediated oxidative stress and inflammation were attenuated by cimifugin via the inhibition of NF-κB/MAPK signaling cascade.

IMQ is a specific agonist of Toll-like receptor (TLR) 7/8, which activates immune cells and dendritic cells, promotes the release of inflammatory cytokines and epidermal hyperplasia [2,21,22]. The topical application of IMQ in mice has been widely used to mimic the psoriatic skin lesions, due to the similar phenotypic and histological symptoms with human psoriasis lesions [23]. Thus, in this work, IMQ cream was topically administrated to induce psoriasis-like animal models. Here, we noticed that IMQ caused severe erythema and scale formation, increased skin thickness, and exacerbated epidermal hyperplasia, which were consistent with previous literature [6,23]. Wang et al. suggested that cimifugin suppressed the ear thickness and histological lesions in atopic dermatitis models [16]. However, the effect of cimifugin on the psoriasis-like lesions is unclear. Our results showed that cimifugin treatment could attenuate the symptoms in psoriasis mice, including decreased PASI scores, smoother skin tissues, and less thickened epidermis. Altogether, these data suggested that cimifugin exerted protective effects against psoriasis-like symptoms.

Increasing evidence reported that oxidative stress was a critical response in diverse skin disorders, such as psoriasis, atopic dermatitis, and vitiligo [6,24]. The generation of oxidative stress may be attributed to the disturbance between reactive oxygen species (ROS) and antioxidants [25]. Generally, the increased ROS production might increase the oxidant factor levels, like MDA and decrease antixodant enzymes, such as GSH, SOD, and CAT [26,27]. Our data showed that IMQ or TNF-α application up-regulated the increase in MDA levels, and down-regulated the decrease in GSH, SOD, and CAT levels in both *in vivo* and *in vitro* models [28]. Nevertheless, cimifugin administration showed an inhibitory effect on the imbalance of oxidant and antioxidant factor production, indicating that antioxidation might be a possible mechanism of cimifugin in psoriasis.

Importantly, oxidative damage might result in the activation of T cells and keratinocytes, as well as the release of proinflammatory cytokines, thus triggering inflammatory responses in psoriasis [29]. Then we also investigated the alterations of proinflammatory cytokines in the present study. Th1 and Th17 cells were suggested to play a critical role in the pathogenesis of psoriasis. Tan et al. showed that the cytokines secreted by Th1 (TNF-α, IFNγ, and IL-2) and Th17 (IL-17A, IL-17F, IL-22, IL-26, and TNF-α) cells were elevated in the serum of psoriasis patients [30]. Previous studies demonstrated that the pro-inflammatory cytokines, including TNF-α, IL-6, IL-1β, IL-17A, and IL-22 were up-regulated in psoriatic skin tissues and serum, and the IL-23/IL-17 axis was found to participate in the regulation of IMQ-induced psoriasis-like skin inflammation [23,31]. Similar to these findings, we observed significant production of proinflammatory cytokines in IMQ-treated mice. In addition, ICAM-1 was an important molecule to recruit immunocytes to the skin and contribute to psoriasis, which could be triggered by TNF-α in diverse cell types [32]. Thus, we also found that, besides IL-6 and IL-1β, ICAM-1 levels were up-regulated in keratinocytes stimulated by TNF-α [33]. Cimifugin administration suppressed the increases in proinflammatory cytokines, which were in accord with previous studies showing the anti-inflammatory effect of cimifugin in atopic dermatitis and rheumatoid arthritis [16,17]. Together, our results suggested that cimifugin might protect against psoriasis-like lesions by inhibiting oxidative stress and inflammation.

It was well-known that oxidative stress might activate essential signaling pathways, such as NF-κB and MAPK, and control relative gene expression [34]. Liu et al. demonstrated that MAPKs participated in the activation of NF-κB signaling pathway in various inflammatory diseases [35]. Both MAPKs and NF-κB signaling cascades had implications in regulating numerous extracellular signals to affect inflammatory responses [36]. Previous studies suggested that MAPKs and NF-κB might trigger inflammatory states, promote epidermal hyperproliferation and exacerbate psoriatic pathogenesis [37,38]. To further unravel the molecular mechanism underlying the anti-oxidant and anti-inflammatory effect by cimifugin in psoriasis, we investigated the role of MAPK and NF-κB signaling cascades in cimifugin-mediated anti-oxidation and anti-inflammation. In the present study, the results indicated that the NF-κB and MAPKs signaling pathways were inhibited by cimifugin in psoriasis-like models, which further demonstrated that the protective effects of cimifugin in psoriasis-like pathogenesis were associated with the inactivation of NF-κB/MAPK. Furthermore, previous studies reported that cimifugin might inhibit allergic inflammation through regulating tight junctions in atopic dermatitis [39], implying that tight junction restoration might be implicated in the possible mechanisms of cimifugin in psoriasis-like pathogenesis.

In conclusion, this current work suggests cimifugin is beneficial for psoriasis-like lesions, which is attributed to its inhibitory effect on oxidative stress and inflammation via inactivating NF-κB/MAPK signaling pathway. These findings may provide a promising and safe agent for psoriasis treatment. However, the animal models used IMQ to mimic psoriasis are partially different from the pathogenesis of psoriasis in humans. Thus, further studies will stay some focuses on the clinical samples to better explore the effect of cimifugin.

## Abbreviations

CAT: catalase
CIM: cimifugin
ELISA: enzyme linked immunosorbent assay
GSH: glutathione
IMQ: imiquimod
MDA: malondialdehyde
PASI: psoriasis area severity index
ROS: reactive oxygen species
SOD: superoxide dismutase
